# The molecular basis for stability of heterochromatin-mediated silencing in mammals

**DOI:** 10.1186/1756-8935-2-14

**Published:** 2009-11-04

**Authors:** Kyoko Hiragami-Hamada, Sheila Q Xie, Alexander Saveliev, Santiago Uribe-Lewis, Ana Pombo, Richard Festenstein

**Affiliations:** 1 Gene Control Mechanisms and Disease Group, MRC Clinical Sciences Centre, Imperial College, Hammersmith Hospital, London W12 0NN, UK; 2 Genome Function Group, MRC Clinical Sciences Centre, Imperial College, Hammersmith Hospital, London W12 0NN, UK; 3 Laboratory for Chromatin Dynamics, Riken Kobe Institute, Centre for Developmental Biology, Kobe, Hyogo 650-0047, Japan; 4 Division of Immune Cell Biology, National Institute for Medical Research, Mill Hill, London NW7 1AA, UK; 5 Epigenetics and Imprinting Laboratory, Department of Oncology, University of Cambridge, CRUK-CRI, Li Ka Shing Centre, Cambridge CB2 0RE, UK

## Abstract

The archetypal epigenetic phenomenon of position effect variegation (PEV) in *Drosophila *occurs when a gene is brought abnormally close to heterochromatin, resulting in stochastic silencing of the affected gene in a proportion of cells that would normally express it. PEV has been instrumental in unraveling epigenetic mechanisms. Using an *in vivo *mammalian model for PEV we have extensively investigated the molecular basis for heterochromatin-mediated gene silencing. Here we distinguish 'epigenetic effects' from other cellular differences by studying *ex vivo *cells that are identical, apart from the expression of the variegating gene which is silenced in a proportion of the cells. By separating cells according to transgene expression we show here that silencing appears to be associated with histone H3 lysine 9 trimethylation (H3K9me3), DNA methylation and the localization of the silenced gene to a specific nuclear compartment enriched in these modifications. In contrast, histone H3 acetylation (H3Ac) and lysine 4 di or tri methylation (H3K4me2/3) are the predominant modifications associated with expression where we see the gene in a euchromatic compartment. Interestingly, DNA methylation and inaccessibility, rather than H3K9me3, correlated most strongly with resistance to de-repression by cellular activation. These results have important implications for understanding the contribution of specific factors involved in the establishment and maintenance of gene silencing and activation *in vivo*.

## Background

Interphase eukaryotic nuclei contain two forms of chromatin [1]: densely DNA-stained regions termed heterochromatin and more diffusely stained regions called euchromatin. In contrast to euchromatin, heterochromatin is rich in repetitive DNA elements, poor in transcriptionally active genes, highly resistant to nuclease digestion and the DNA replicates late in S-phase [2]. In mammals, constitutive heterochromatin is enriched with specific chromatin modifications including histone H3 lysine 9 (H3K9) trimethylation (me3) [3-5], H4K20me3 [6-8] and DNA methylation [9-12], all of which have been implicated in gene silencing. These modifications may occur in a coordinated manner. For instance, mice deficient in Suv39h, a H3K9 histone methyltransferase (HMTase), have reduced DNA methylation at their pericentric repeats [11], indicating the interdependence between these modifications. Furthermore, the relationship between H3K9me3 and DNA methylation has been implicated in the regulation of genes involved in early development and across species [13,14].

The gene-repressive effect of heterochromatin was first demonstrated by position effect variegation (PEV) [15] in *Drosophila*, where a normally euchromatic *white+ *gene (responsible for red eye pigmentation) was silenced in a proportion of eye cells when the gene was placed abnormally close to a block of pericentric heterochromatin. Similar phenomena were observed in organisms ranging from yeast to mice when a reporter gene was inserted within pericentric or telomeric regions of chromosomes [16-18]. The extent of heterochromatin-induced silencing effects can be modulated by the dosage of chromatin modifiers. This was elegantly shown in the *Saccharomyces cerevisiae *telomere position effect (TPE)[19,20]. In addition, mutations in genes encoding homologues of Suv39 [17,21-23] or a structural chromatin component [24-27], heterochromatin protein 1 (HP1) [22,28-30], led to reduced silencing of variegating reporter genes in *Drosophila *and *Schizosaccharomyces pombe*. On the other hand, enhanced/increased silencing of a variegating reporter gene was observed with over-expression of Su(var)3-9 [21] or of an HP1 homologue in *Drosophila, S. pombe *and mice [29,31,32].

Biochemical analyses in *Drosophila *and murine PEV revealed that repressed variegating genes have a chromatin structure and/or nucleosome organization pattern comparable to constitutive heterochromatin of pericentric regions [18,33,34]. Consistent with the spreading hypothesis for heterochromatin formation, chromosomal rearrangement affecting two reporter genes resulted in silencing of the reporter proximal to the rearrangement breakpoint whenever the reporter distal to the breakpoint was silenced [35,36]. This was accompanied by 'compaction' visualized as the darkening of polytene chromosome bands adjacent to the rearrangement breakpoint, suggesting the spreading of heterochromatin over the breakpoint. Based on these earlier observations, together with the sensitivity of PEV to the dosage of chromatin modifiers, it has been thought that PEV results from the stochastic spreading of heterochromatin-forming factors or heterochromatic chromatin marks from a nearby heterochromatic region into the variegating gene, in the absence of dominant *cis*-acting boundary elements [37-39]. The spreading of heterochromatin may occur in a linear manner or in *trans*, where an interaction between a variegating gene and heterochromatin on the same or another chromosome occurs [40,41]. In a landmark study, Harmon and Sedat demonstrated the correlation between silencing in *Drosophila *PEV and the localization of the reporter to heterochromatin [41]. However, there is little molecular evidence for the spreading of 'heterochromatic' chromatin modifications over a repressed variegating gene in vertebrates.

Here, we investigated the chromatin modifications associated with a variegating transgene and dissected out their roles in chromatin compaction and stable gene silencing, using the variegating human CD2 (hCD2) transgenic mouse system [18,31]. Our results revealed that repressed variegating hCD2 transgenes are indeed associated with known heterochromatic chromatin modifications, including H3K9me3 and DNA methylation, and positioned within or close to a repressive nuclear domain. However, DNA methylation was the key modification that accompanied the formation of an inaccessible chromatin structure and more stable gene silencing upon cellular activation and through cell division.

## Results

### hCD2 transgenic mice as a model for mammalian PEV

**Figure 1:**
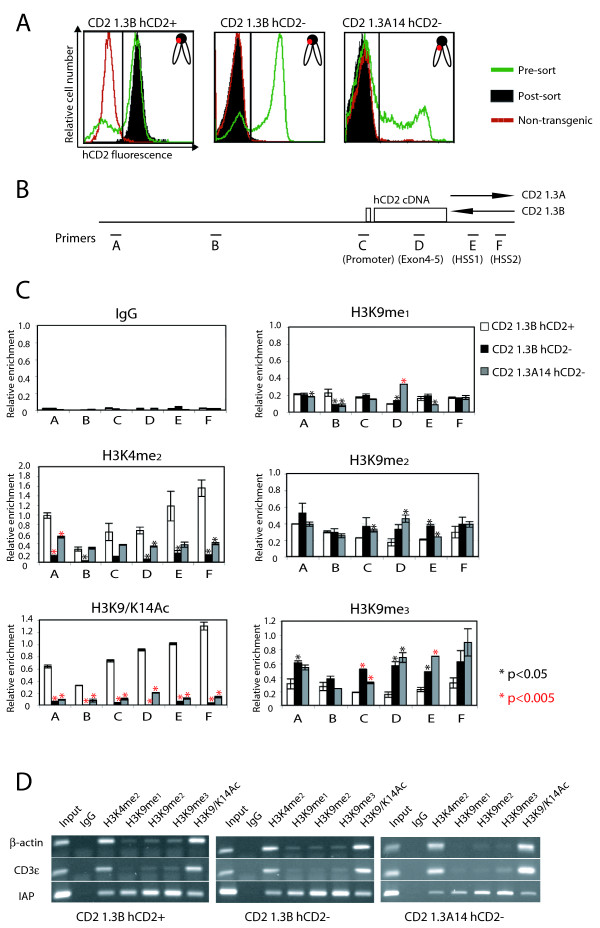
**Histone modification patterns of expressed and repressed hCD2 transgene in the CD2 1.3 variegating transgenic lines**. (A) fluorescence-activated cell sorter (FACS) analysis of hCD2 expression on the surface of pre-sorted (green) and sorted (solid black) peripheral T cells. The hCD2 expression profile of non-transgenic T cells is shown in red. Chromosomal location of hCD2 transgene (orange circle) in each transgenic line is shown in the plots: pericentric regions and chromosome arms are shown as a black circle and white ovals respectively. (B) Schematic diagram of hCD2 transgene locus. Note that the 3' regulatory region is oriented in reverse directions in the CD2 1.3B and CD2 1.3A14 transgenic line. Locations of the primers used for chromatin immunoprecipitation (ChIP) assays are indicated with black bars and letters at the bottom. (C) ChIP analysis of histone modifications along the hCD2 transgene in hCD2+ and hCD2- T cells. ChIP was performed with chromatin prepared from sorted hCD2+ or hCD2- T cells from CD2 1.3B (white or black bar) and CD2 1.3A14 (grey bar) transgenics using antibodies against various histone H3 modifications. Enrichment for each modification was determined by qPCR and normalized to 5% input (black asterisks = *P *< 0.05, red asterisks = *P *< 0.005). This experiment was repeated three times (error bars = standard deviation). (D) control PCR on ChIP-ed materials using primers against expressed (CD3ε, β-actin) or repressed (IAP) loci.

In order to investigate chromatin modifications associated with a repressed hCD2 transgene, we used the CD2 1.3B and CD2 1.3A14 variegating mouse lines that have been previously described [31]. Both these transgenic lines carry an hCD2 transgene with a truncated locus control region (LCR - which is known to be necessary for chromosomal position-independent expression of the transgene) and have been shown to exhibit variegated expression of hCD2 protein on the surface of T cells, irrespective of the orientation of the truncated LCR [18]. The CD2 1.3B transgenic line contains six copies of hCD2 transgenes integrated within a block of pericentric major satellite repeats. The CD2 1.3A14 transgenic line, on the other hand, carries approximately 14 copies of the transgene integrated close to, but outside, the pericentric heterochromatin [31]. As seen with PEV in other organisms, enhanced variegation was observed in these transgenic mice in response to HP1β over-expression [31]. The following analyses were performed on sorted hCD2 expressing (hCD2+) and/or hCD2 non-expressing (hCD2-) T cells isolated from mesenteric lymph nodes and spleens of CD2 1.3B and CD2 1.3A14 transgenic mice (Figure 1A). hCD2+ T cells from non-variegating CD2-LCR (called here, minigene 4 (MG4)) transgenic mice [31] were also used as controls for some analyses. It should be noted that the level of surface hCD2 protein was previously shown to correlate with hCD2 mRNA level [42].

### Repressed hCD2 transgenes exhibit histone modification patterns similar to pericentric heterochromatin

Pericentric heterochromatin is enriched with H3K9me3 and contains few modifications associated with transcriptional activation such as H3K9/K4Ac or H3K4me2/3. In order to test whether repressed hCD2 transgenes have a histone modification pattern comparable with pericentric heterochromatin, we performed chromatin immunoprecipitation (ChIP) on chromatin prepared from sorted hCD2+ and/or hCD2- T cells from CD2 1.3B and 1.3A14 transgenics, using antibodies against various histone modifications indicated in Figure 1C. Enrichment for histone modifications along the 8kb hCD2 transgene locus was analysed by quantitative polymerase chain reaction (PCR) using primer pairs for six different regions of the transgene (Figure 1B). Controls were the β-actin and CD3ε genes, which are transcribed in T cells, and the 5' long terminal repeats (LTR) of intracisternal A particle (IAP), which is a transposon element present in many repressed copies in the murine genome. For each transgenic line, β-actin and CD3ε showed enrichment for H3K4me2 and H3K9/K14Ac whereas IAP was mainly enriched with H3K9me2/3, as anticipated (Figure 1D). hCD2 transgenes in hCD2- T cells from both CD2 1.3B and CD2 1.3A14 transgenic mice were enriched with H3K9me3 and were markedly depleted in H3K4me2 and H3K9/K14Ac (Figure 1C). In contrast, hCD2 transgenes in hCD2+ T cells showed high levels of H3K4me2 and H3K9/K14Ac (Fig. 1C). H3K9me1/2 was detected at the transgene with similar levels in hCD2+ and hCD2- T cells (Figure 1C). These modifications are not predominant marks for pericentric heterochromatin [3-5]. Thus, the repressed hCD2 transgene in both CD2 1.3B and CD2 1.3A14 transgenics have a histone modification pattern similar to that of pericentric heterochromatin. However, levels of H3K4me2 and H3K9/K14Ac were much lower along the repressed transgenes in CD2 1.3B than those in CD2 1.3A14 transgenic mice (Additional file 1, Figure S3A). In CD2 1.3A14 transgenic mice, the H3K9me3 mark was preferentially enriched at the coding and 3' regulatory region, compared with the distal and proximal promoter regions (Additional file 1, Figure S3B). This suggests that the transgene in CD2 1.3B and CD2 1.3A14 transgenic mice may be repressed by a mechanism involving H3K9me3, but the mode of acquisition or maintenance of this mark may differ between the two lines, as may the nucleosome occupancy which would be interesting to assess in more detail.

### Key regulatory regions of repressed hCD2 transgenes are marked by DNA methylation

**Figure 2:**
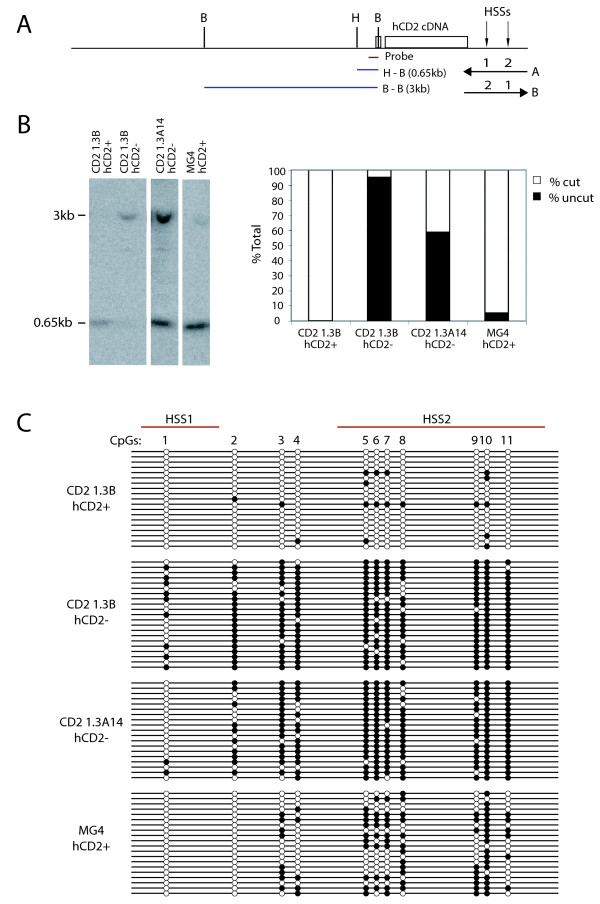
**The DNA methylation status of the hCD2 transgene in hCD2+ and hCD2- T cells from the CD2 1.3 transgenic lines**. (A) Schematic transgene map showing the restriction sites for *Bgl*II (*B*) and *Hha*I (*H*) and location of the probe used in B. (B) The CpG methylation analysis of the proximal promoter region by methylation-sensitive restriction enzyme digest and Southern blot. The bar charts show the ratios between the unmethylated and methylated *Hha*I sites. (C) Bisulfite sequencing analysis of the 3' regulatory regions of the hCD2 transgene. Methylated and unmethylated CpGs are shown as filled and open circles, respectively. Each line represents the sequence from a single clone. Similar results were obtained from two independent experiments. Comparisons between numbers of methylated CpGs were done using the Mann-Whitney U-test and Fisher's exact test (see text).

Despite its role in other forms of epigenetic silencing, such as genomic imprinting, in mammals little is known about the involvement of DNA methylation in PEV. However, mammalian DNA methyltransferases have been reported to localize at pericentric heterochromatin and can be recruited to chromatin through known PEV modifiers such as HP1 and Suv39h1 [11,43]. Therefore, we next investigated whether DNA methylation is associated with repressed hCD2 transgenes by analysing the DNA methylation pattern at the promoter and enhancer regions of the hCD2 transgenes. The promoter of the hCD2 transgene contains two CpGs approximately 100 bp upstream of the transcriptional start site (Figure 2A). DNA methylation at the CpGs was tested by digestion with a methylation-sensitive restriction enzyme, *Hha*I and Southern blot (Figure 2B). The promoter CpGs were almost completely unmethylated (as indicated by 95%-100% digestion by *Hha*I) in hCD2+ T cells from CD2 1.3B and MG4 transgenic mice (Figure 2B). Notably, the promoter CpGs were highly resistant to digestion with *Hha*I in hCD2- T cells from CD2 1.3B and CD2 1.3A14 transgenic mice (about 5% and 40% digestion, respectively), indicating a high level of DNA methylation at the promoter CpGs of repressed hCD2 transgenes (Figure 2B). In contrast to the promoter, the enhancer region of the hCD2 transgene is relatively CpG-rich. As shown in Figure 2C, there are 11 CpGs, encompassing a region of 800 bp containing the 3' end of DNase I hypersensitivity site (HSS) 1 (CpG site 1) and all of HSS2 (CpG site 5-11). HSS1 has previously been shown to act as an enhancer [44]. DNA methylation status of the 11 CpGs was analysed by metabisulfite sequencing. Cytosine in most of the 11 enhancer CpGs in hCD2- T cells was methylated at a high frequency, whereas, in hCD2+ T cells, a large proportion of unmethylated cytosines were observed at the enhancer CpGs (Figure 2C, *P *= 0.0002 by the Mann-Whitney's U-test). This indicates that, similar to DNA methylation at the promoter, the enhancer region of repressed hCD2 transgenes is highly methylated. Thus, we show here that DNA methylation strongly correlates with gene silencing associated with mammalian pericentric PEV. Interestingly, the most marked difference in DNA methylation level among different hCD2- T cell populations was observed at CpG sites 1 and 2, which lie within or proximal to HSS1. In contrast to other enhancer CpGs, DNA methylation at CpG sites 1 and 2 in hCD2- T cells was markedly reduced in the CD2 1.3A14 transgenic mice compared to these sites in the CD2 1.3B mice (Figure 2C, *P*_HSS1/HSS2 _<0.05 by Fisher's exact test). Hence, in addition to the difference in H3K9me3 marking (Figure 1C), the pattern and level of DNA methylation along repressed hCD2 transgenes also seems to differ between the two transgenic lines.

### A repressed hCD2 transgene is preferentially located at a repressive nuclear domain

**Figure 3:**
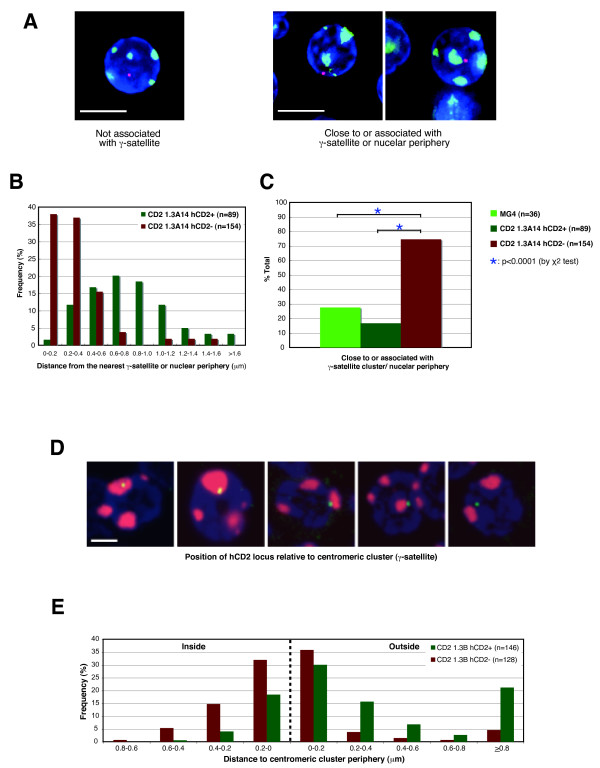
**Nuclear localization of the hCD2 transgene in hCD2+ and hCD2- T cells**. (A, B, C) Three-dimensional fluorescence in *in situ *hybridization (FISH) analysis of the hCD2 transgene location in the nuclei of CD2 1.3A14 and MG4 transgenic T cells. (A) Deconvoluted images showing typical examples of two nuclear localization patterns of the hCD2 transgene (red) relative to γ-satellite clusters (green) and to the nuclear periphery (determined by DAPI-staining, blue). Bar: 5 μm. (B) The distance between the hCD2 transgene signal and the nearest γ-satellite clusters or nuclear periphery in CD2 1.3A14 hCD2+ and hCD2- T cells. The distance was measured from the centre of the transgene signal to the edge of a pericentric cluster or nuclear periphery. Median for hCD2+ T cells = 0.76 μm, that for hCD2- T cells = 0.29 μm. The difference in the distance of the transgene signal from heterochromatin between hCD2+ and hCD2- T cells was statistically significant: *P *< 0.0001 by K-S test. (C) The percentage of hCD2+ and hCD2- T cells from the indicated transgenic lines that show close proximity of the transgene signal to heterochromatic nuclear compartments. The difference in the transgene location between hCD2+ and hCD2- T cells was statistically significant (*P *< 0.0001 by χ^2 ^test). Note that as expected the difference in the transgene location between Mg4 and CD2 1.3A14 hCD2+ T cells was not statistically significant (*P *> 0.05 by χ^2 ^test). (D) The position of the hCD2 locus relative to the centromeric cluster was determined in sorted CD2+ and CD2- T cells, by cryoFISH using Rhodamine-labelled γ-satellite for detection of the centromeric cluster (red) and the DIG-labelled hCD2-cos1 cosmid probe to detect hCD2 loci (green). Nucleic acids were counterstained with TOTO-3 (blue). Bar: 2 μm. (E) The frequency of association of hCD2 locus with centromeric cluster were measured from the centre of the hCD2 signal to the periphery of the γ-satellite signal.

The location of a gene within the nucleus has been implicated in the regulation of its activity. In mammalian cells, pericentric heterochromatin and the nuclear periphery have been regarded as repressive nuclear domains [45,46]. To test whether the repressed state of the hCD2 transgene correlates with its association with the repressive nuclear domains, three-dimensional (3D) fluorescence in *in-situ *hybridization (FISH) was undertaken on sorted hCD2- and hCD2+ T cells from CD2 1.3B, 1.3A14 and MG4 transgenic mice. The pattern of hCD2 transgene localization was divided into two categories: (1) not associated with the heterochromatic major satellite DNA nor nuclear periphery; and (2) close to, or associated with, the major satellite DNA or nuclear periphery (as determined by 4',6-diamidino-2-phenylindole (DAPI) counter-stain) (Figure 3A). The distance between the centre of a transgene signal and the edge of the nearest major satellite cluster or nuclear periphery was measured. hCD2+ and hCD2- T cells from CD2 1.3A14 mice showed different distributions of the transgene signal relative to the nuclear periphery and a pericentric cluster (Figure 3B, comparisons of the distributions using the non-parametric Kolmogorov-Smirnov test, *P *< 0.0001). The transgene in hCD2- T cells from CD2 1.3A14 transgenic mice was found less than 0.4 μm from either the nuclear periphery or the major satellite repeat (whichever was the closer) in approximately 75% of cells (Figure 3C, for the details see Additional file 2, Table S3). In striking contrast, the hCD2 signals in hCD2+ T cells from this transgenic line were not associated with the major satellite and localized away from the nuclear periphery in over 80% of cells (Figure 3C). Similar percentages of hCD2 signal distribution were observed with hCD2+ T cells from the MG4 transgenic mice (approximately 70% not associated with the major satellite or nuclear periphery, Figure 3C). Hence, in these transgenic mouse lines, the nuclear localization of the hCD2 transgene correlates with its expression status. With the CD2 1.3B transgenic line, in which the transgene is integrated within the major satellite repeat, the nuclear localization pattern of the hCD2 transgene also differed in expressing and silent cells. The transgene signal was observed at the periphery of a major satellite cluster in most hCD2+ T cells from CD2 1.3B transgenic mice (data not shown), whereas a transgene signal in hCD2- T cells from the mice was undetectable by our 3D method. One possibility was that the inability to detect the hCD2 transgene signal might be due to the inaccessibility of the transgene locus to the probe. To overcome this problem, and also to analyse the transgene location in the CD2 1.3B transgenic line at a higher resolution, we performed FISH on ultrathin (approximately 150nm thick) sections (cryo-FISH) of FACS-sorted hCD2+ and hCD2- T cells from the transgenic mice (Figure 3D and 3E) [47]. First, we tested the differences between the two distributions of distance of the locus to the periphery of the nearest centromeric cluster (Figure 3E). The hCD2 loci were more distant in the hCD2+ than the hCD2- cells (comparisons of the distributions using Kolmogorov-Smirnov test, *P *< 0.001). When we compared the number of loci that were separated from the periphery, we found that 47% of hCD2 loci were >0.2 μm from the periphery of centromeric clusters in hCD2+ cells compared to 11% in hCD2- cells (Chi-squared test, *P *< 0.0001). Moreover, in 53% of hCD2- T cells the hCD2 signal was observed within a major satellite cluster compared to 23% in hCD2+ cells (Chi-square test, P < 0.0001,). Taken together, the repressed hCD2 transgene in the CD2 1.3B transgenic line was more closely associated with the major satellite cluster, whereas the expressed counterpart appeared to be more frequently 'flipped out' from a major satellite cluster and positioned at the surface of, or away from, the large block of pericentric heterochromatin. This is reminiscent of observations made with the pericentrically integrated λ5 transgene [48].

### Absence of the promoter but not the enhancer DNase I hypersensitivity correlates with the repressed status of the hCD2 transgene

**Figure 4:**
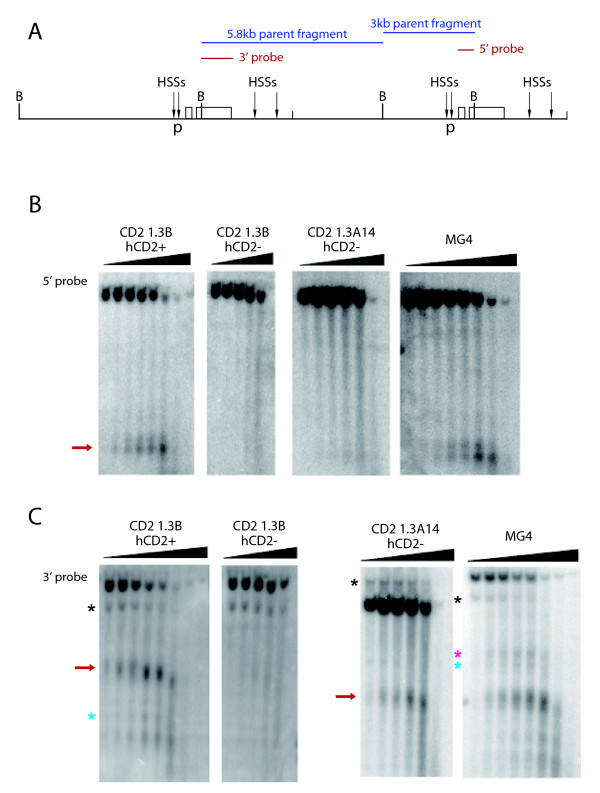
**DNase I hypersensitivity analysis on hCD2+ and hCD2- T cell nuclei from CD2 1.3B, CD2 1.3A14 and MG4 transgenic mice**. (A) Transgene map showing locations of promoter (p) and enhancer HSSs, the restriction sites for *Bgl*II (*B*) and the probe locations. DNase I HSS at the hCD2 promoter (B) and enhancer (C) are indicated by red arrows. Black, blue and pink asterisks indicate transgene end-fragments, HSS2 and HSS3, respectively.

We next investigated whether the chromatin modifications described above directly correlate with formation of a higher-order chromatin structure over the hCD2 transgene locus. The extent of chromatin compaction was tested by measuring the accessibility of DNase I to promoter and enhancer regions of the hCD2 transgene [18,42,44] in nuclei of sorted hCD2+ and hCD2- T cells (Figure 4A). The promoter HSS was only detected in nuclei of hCD2+ T cells from CD2 1.3B and MG4 but not in hCD2- T cells from both CD2 1.3B and 1.3A14 transgenic mice. This is consistent with previously published data from other variegating hCD2 transgenics [18,31,42] (Figure 4B). Thus, the promoter HSS correlates with the expression status of the hCD2 transgene. On the other hand, the enhancer HSS (HSS1) was detected in the nuclei of both hCD2+ T cells and hCD2- T cells from CD2 1.3A14 transgenic mice (Figure 4C). Only in nuclei of hCD2- T cells from CD2 1.3B transgenic mice were both the promoter and enhancer markedly resistant to DNase I digestion (Figure 4B, C). The high accessibility of HSS1 to DNase I in hCD2- T cells from the CD2 1.3A14 line implied unstable nucleosomes and was rather surprising, as the region was enriched with H3K9me3 which is normally found in inaccessible regions (Figure 1C). The difference in the accessibility of the enhancer HSS in hCD2- T cells between CD2 1.3B and 1.3A14 transgenic mice appears to correlate closely with the level of DNA methylation at the region (Figure 2C). Taken together, opening of the promoter and enhancer HSSs may be necessary for expression of the hCD2 transgene but repression of the hCD2 transgene can be achieved by compaction of the promoter HSS alone, irrespective of the presence or absence of the enhancer HSS. Moreover, in combination with results from the ChIP and bisulfite sequencing assays, compaction of the enhancer HSS in this case might be modulated by DNA methylation rather than H3K9me3.

### The stability of hCD2 transgene repression correlates with chromatin conformation at the enhancer

**Figure 5:**
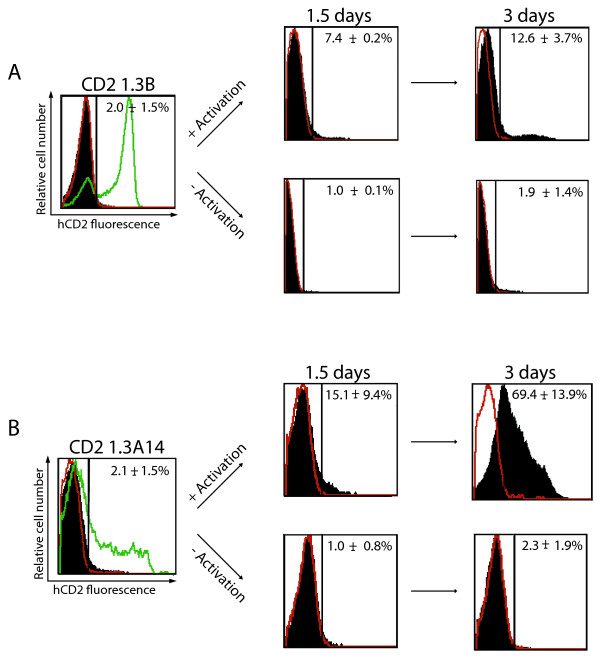
**Analysis of T cell activation-induced hCD2 derepression in hCD2- T cells from CD2 1.3B and CD2 1.3A14 transgenic mice**. The cells were activated for 1.5 and 3 days by TCRβ/CD28 cross-linking. Changes in the surface expression of hCD2 were monitored by FACS: pre-sort (green), post-sort (solid black) and non-transgenic (red). Accompanying numbers in FACS plots indicate the percentage of hCD2 expressing or derepressed T cells (± standard deviation).

The classical definition of *Drosophila *PEV implies clonal heritability of an on/off state of a variegating gene. However, a few previous studies have described some instability of heterochromatic silencing in PEV and TPE [17,48-50]. In fact, derepression of the hCD2 transgene was observed in some T cells from CD2 1.3B transgenic mice through cell divisions [51]. Hence, we took advantage of this ability of an hCD2 transgene to derepress upon T cell activation/proliferation to investigate whether the pre-acquired chromatin modifications and chromatin conformation (at the enhancer) might affect the stability of hCD2 transgene repression. hCD2- T cells sorted from CD2 1.3B and 1.3A14 transgenics were activated by T cell receptor β (TCRβ) and CD28 co-stimulatory molecule cross-linking for 1.5 or 3 days and changes in surface expression of hCD2 protein were monitored by FACS (Figure 5). For both the CD2 1.3B and CD2 1.3A14 transgenic lines, derepression of the hCD2 transgene was observed with prolonged T cell activation, but the extent of hCD2 derepression differed greatly between the two transgenic lines. After 1.5 days of activation, the derepression of the hCD2 transgene was observed in around 7.4% of hCD2- T cell population from CD2 1.3B transgenic mice (Figure 5A). The percentage of the hCD2-derepressed population almost doubled (12.6 ± standard deviation [SD] 3.7%) after a further 1.5 days of activation. The proportion of hCD2-derepressed T cells was very similar to that described previously [51]. In contrast, hCD2- T cells from CD2 1.3A14 transgenic mice exhibited much more derepression of the hCD2 transgene (Figure 5B). At the earlier time point, approximately 15% ± SD 9% of the hCD2- T cell population from the transgenic mice derepressed the hCD2 transgene but the proportion of hCD2-derepressed T cells dramatically increased to approximately 70% ± SD 14% after 3 days of TCRβ/CD28 cross-linking. For both transgenic lines, no marked hCD2 derepression in hCD2- T cells was observed without TCRβ/CD28 cross-linking (Figure 5A, B). Moreover, as the extent of T cell activation and cell proliferation of the hCD2- T cell population were comparable between the CD2 1.3B and CD2 1.3A14 transgenic line (Additional file 3, Figure S1), the difference in the degree of hCD2 derepression reveals a difference in the susceptibility of the repressed hCD2 transgene to derepression between these two lines. The greater susceptibility of repressed hCD2 transgenes to derepression in the CD2 1.3A14 transgenic line correlates with the presence of the enhancer HSS and compromised repressive chromatin 'marking' described earlier. In support of this, a similar instability of hCD2 transgene silencing was also observed with CD2 1.3-CTG transgenic mice [42] and another CD2 1.3 transgenic mouse line where hCD2- T cells exhibited high accessibility to DNase I at the enhancer and extensive hCD2 derepression upon T cell activation (Additional file 4, Figure S2 and data not shown). In comparison with a study on the endogenous *Dntt *locus [52], it is possible that the relatively long-range spreading of chromatin marks and/or a compaction of chromatin along the hCD2 transgene locus may be necessary for the stable repression of the transgene. Taken together, our results suggest that the CD2 1.3A14 and 1.3B transgenics differ in the detailed molecular mechanisms for hCD2 repression, suggesting further complexity of the mechanisms involved in 'heterochromatin-mediated' silencing [53].

## Discussion and conclusion

### Epigenetic differences between expressed and repressed hCD2 transgenes

**Figure 6:**
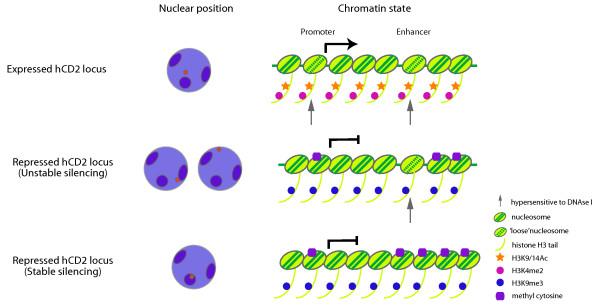
**Integrating nuclear position, histone modifications and accessibility of the gene in maintaining heterochromatin-mediated gene silencing *in vivo***. Different 'epigenotypes' (right) are depicted alongside the expression status (left) of the affected gene - DNA methylation and locus inaccessibility were found to have the strongest correlation with stable gene silencing.

This report identifies specific chromatin marks associated with an expressed and repressed variegating gene *in vivo/ex vivo *in mammalian cells and correlates this with nuclear localization of the repressed gene. Unlike previous studies on *Drosophila *PEV, the use of hCD2+ and hCD2- T cells from the individual CD2 1.3B transgenic mice enabled us to examine 'pure' epigenetic differences between the expressed and repressed hCD2 transgene. Consistent with the previously observed sensitivity of PEV to Suv3-9 homologues [17,21-23], repressed hCD2 transgenes in both CD2 1.3B and CD2 1.3A14 transgenic mice were associated with H3K9me3 whereas expressed hCD2 transgenes were enriched with 'euchromatic' modifications such as H3K4me2 and H3K9/K14Ac. We also demonstrated that repressed hCD2 transgenes are embedded within, or positioned close to, a repressive nuclear domain whereas expressed transgenes are located away from such a domain. Moreover, despite a previously reported lack of correlation between DNA methylation and variegation of a reporter gene in mice [54], our results show that the relative abundance of DNA methylation at the regulatory regions of a hCD2 transgene reflects the on/off state of the hCD2 transgene. These observations suggest that variegation of hCD2 transgenes involves multiple layers of epigenetic regulation (Figure 6).

### Distinct roles of H3K9me3 and DNA methylation in hCD2 transgene silencing

The differences in the levels/patterns of repressive chromatin modifications/chromatin compaction along the repressed hCD2 transgene and the stability of hCD2 repression in two different hCD2 transgenic lines (CD2 1.3A14 and CD2 1.3B) suggested an unequal contribution of DNA methylation and H3K9me3 to maintenance of hCD2 repression. Although, we have not yet studied nucleosomal occupancy directly, our results suggest that a higher level of DNA methylation rather than H3K9me3, correlates with compaction of the hCD2 regulatory regions and more stable hCD2 repression. This is consistent with recent studies which indicate that DNA methylation appears to prevent reactivation of GFP transgenes [55] and developmentally regulated genes [56] in mammalian cells. However, it must be noted that the H3K9 HMTase, Suv39h, is an important factor in the establishment of repression of the hCD2 transgene (S Uribe-Lewis, unpublished data). Furthermore, it will be interesting to study other histone modifications such as H3K27me3 and H4K20me3 in the future. Taken together, different chromatin modifications might contribute differentially during the initiation and maintenance stages of hCD2 repression.

### Open enhancer conformation and instability of hCD2 silencing

Conformation of the enhancer chromatin appears to correlate with the stability of hCD2 repression during T cell activation (Figure 4 and 5). It is possible that the open enhancer conformation in CD2 1.3A14 transgenic mice might lead to greater hCD2 derepression as *trans*-activators, which become up-regulated during T cell activation, may bind to the enhancer and promote expression of a previously repressed hCD2 transgene. Alternatively, insertion into the major satellite sequences might lead to an increased spreading of the heterochromatic state into the CD2 1.3B transgene making it more resistant to derepression than the CD2 1.3A14 transgene array, which integrated near to pericentric heterochromatin. Another possibility is that the CD2 1.3A14 array is more subject to the effect of transgene copy number whereas the pericentric array is affected more by the satellite repeats [57]. Furthermore, in accordance with recent descriptions of H3K9 demethylases [58-60], previously acquired H3K9me3 at the enhancer may not be enough to keep the hCD2 transgene in a repressed state if an H3K9 demethylase is recruited to the region by an activator complex. In the case of CD2 1.3B transgenic mice, compaction of the enhancer (and promoter) might prevent the binding of *trans*-activators and further recruitment of activating chromatin modifiers making the hCD2 transgene more resistant to derepression. In addition, as revealed by cryo-FISH, embedding the repressed hCD2 transgene within the highly heterochromatic environment in this transgenic line might maintain a local abundance of heterochromatic factors high, which competitively prevent binding of *trans*-activators to the transgene. However, even in the CD2 1.3B transgenic line, the maintenance of hCD2 transgene repression is incomplete and significant hCD2 derepression occurs with prolonged T cell activation. Interestingly, it has been reported that over-activation of the JAK signalling pathway counteracts heterochromatic silencing [61,62]. As the constitutive heterochromatin is not completely inaccessible to molecules and is maintained by dynamic processes [63-66], the continuous presence of a high level of *trans*-activators might eventually compete with heterochromatic factors and gain access to binding sites on the hCD2 transgene to activate hCD2 expression [67]. Future work will address the role of a growing number of histone modifications that may also participate in these processes. In conclusion, our studies on mammalian PEV have enabled us to dissect *ex vivo *potential epigenetic factors associated with maintenance of gene silencing through cell division and indicate that DNA methylation correlates most strongly with both the maintenance of inaccessibility and the transmission of gene-silencing information to the daughter cell population.

## Materials and methods

### Mice

All hCD2 transgenic mice lines were generated as previously described [18,31]. Transgenic mice that are heterozygous for the hCD2 transgene were used for all the experiments. The project was given ethical approval by Imperial College London and the UK Home Office.

### Cell sorting

Peripheral lymphocytes were obtained from mesenteric lymph nodes and spleens of transgenic mice. T cells were isolated from the mixed population using Dynal T cell negative selection kit (Dynal, UK). hCD2+ T cells were obtained by positive selection using MACS hCD2 microbeads (Miltenyi Biotec). The flow-through containing hCD2- T cells was further purified by incubation with Dynal hCD2 magnetic beads (Dynal, UK) to deplete any contamination with hCD2+ T cells. Alternatively, peripheral T cells were stained with an antibody cocktail containing phycoerythrin (PE)-conjugated anti-hCD2 (BD Pharmingen), tri-colour (TC)-conjugated anti-CD4 and anti-CD8 antibodies (Caltag), and sorted using a fluorescence-activated cell sorter (FACS) DIVA (Becton Dickenson).

### Cell culture

For cell culture, all the sorting procedures were carried out under sterile conditions. Sorted hCD2- peripheral T cells were resuspended in T-cell medium (Iscove's Modified Dulbecco's Medium/10% fetal calf serum [Sigma Aldrich]/1% penicillin/streptomycin/0.05% β-mercaptoethanol/20 U/ml recombinant IL-2 [Roche], all from GIBCO, unless indicated). For T-cell stimulation, 96-or 24-well plates were coated with 10 μg/ml anti-TCRβ chain and 2 μg/ml anti-CD28 co-stimulatory molecules (BD Pharmingen) in phosphate buffered saline (PBS). Cells were cultured at 1 × 10^6 ^or 4 × 10^6 ^cells/well in 96- or 24-well plates, respectively with or without T cell stimulation. To measure the extent of cell proliferation, cells were loaded with 1 μM carboxyfluorescin diacetate succinimidylester (CFSE; Molecular Probes) in PBS prior to the culture.

### FACS analysis

For *ex vivo *T cells, 1 × 10^5 ^cells were stained with PE-conjugated anti-hCD2 (1/200) and TC-conjugated anti-CD4 or CD8 (1/400) antibodies. For cultured T cells, 1 × 10^6 ^T cells were stained with PE-conjugated hCD2, biotin-conjugated anti-CD69 (1/100) and allophycocyanin (APC)-conjugated CD4 or CD8 (1/100; all from BD Pharmingen) or with PE-conjugated hCD2, TC-conjugated CD4/CD8 and APC-conjugated CD25 (1/100; BD Pharmingen). For CD69 staining, cells were incubated with cychrome-conjugated streptavidin (1/400; BD Pharmingen) after staining with the primary antibody. Acquisition and analysis were carried out using FACS Calibur and Cell Quest software (Becton Dickinson). The cut-off point of hCD2 expression level was set according to hCD2 fluorescence of CBA/Ca; >98% of T cells from the negative control fell below the cut-off point.

### ChIP

ChIP was performed on sonicated chromatin prepared from sorted T cells using standard protocols. Chromatin was prepared from 5 × 10^6 ^sorted T cells. Cells were fixed with 1% formaldehyde in X-linking buffer (50 mM 4-(2-hydroxyethyl)piperazine-1-ethanesulfonic acid (HEPES) pH8.0, 100 mM NaCl, 1 mM ethylenediaminetetraacetic acid [EDTA], 0.5 mM ethylene glycol tetraacetic acid [EGTA]) for 5 min. The X-linking reaction was quenched by addition of 125 mM glycine/PBS. This was followed by cell lysis and nuclear lysis in appropriate buffers. X-linked chromatin was then sonicated on ice to produce 300-500 bp chromatin fragments. For each immunoprecipitation (IP) sample, 2 μg chromatin was diluted to a final volume of 375 μl in ice-cold IP buffer (1.1% Triton-X100, 0.01% SDS, 1.2 mM EDTA, 167 mM NaCl, 20 mM Tris-HCl at pH8.1, 10 mM Na butyrate, 0.1 mM phenylmethanesulphonylfluoride, 0.1 mM Benzamidine, 1/1000 Protease inhibitors cocktail [Sigma-Aldrich, UK]) and was incubated with 10 μg anti-dimethyl histone H3K4 (Abcam), 10 μg anti-acetyl histone H3 (Upstate Biotechnology), 10 μg normal rabbit IgG (Santa Cruz) or 5 μg anti-mono-, di- or tri-methyl histone H3K9 (from Dr T Jenuwein) for 3 h at 4°C on a rotator. 100 μl BSA-blocked protein A-agarose beads (Upstate Biotechnology) was added to each tube and incubated for further 1.5 h as before. After the IP, the beads were washed twice each with high salt, low salt and lithium chloride wash buffers. The washed beads were resuspended in an elution buffer and heated at 65°C for 30 min. Eluates were applied to spin-X columns (Fisher Scientific) and spun at 6 k rpm for 2 min to remove the protein A beads. Input and bound fractions were treated with RNase A and proteinase K (both from Roche). IP-ed DNA was purified using Qiagen PCR clean-up kit (Qiagen, UK). Purified IP-ed DNA was diluted five times and purified input DNA was diluted 100 times in sterile H_2_O. For PCR with 'control' primers (IAP, β-actin and CD3ε), the reaction was performed with 5 μl DNA, 1× PCR buffer (Sigma), 2 mM MgCl (Sigma), 0.4 mM dNTPs (Fermentas), 0.2 μM each forward and reverse primer and 2.5 U Taq polymerase (Sigma) in a total volume of 20 μl. The PCR cycle was run as following: the initial incubation at 94°C for 5 min, 28 cycles (IAP) or 40 cycles (β-actin and CD3ε) of denaturing (94°C for 40 s), annealing (60°C for 40 s) and extension (72°C for 36 s) and the final extension at 72°C for 10 min. The PCR products were analysed by electrophoresis of the products on 1.5% TBE agarose gels. For real-time PCR analysis using hCD2 primers, the reaction was carried out with 4 μl DNA, 0.4 μM each forward and reverse primer and 1× SYBR Jump-Start reaction mix (Sigma) in a total volume of 20 μl. PCR cycles were set on and run through Opticon 1 or Opticon 2 program (MJ Research) connected to a PCR cycler. The PCR cycle consisted of the initial activation of Taq polymerase at 94°C for 2 min, 40 cycles of denaturing (94°C for 30 s), annealing (60°C for 30 s) and extension (72°C for 30 s). The fluorescence intensity was read at 75, 78, 80 and 82°C after each cycle. The analysis of resulting C (t) values was performed using the Opticon1/2 program. Threshold was set at 0.1, at which PCR amplification curves were still in a linear range on a logarithmic scale. C(t) values for samples and those for serial dilutions of input DNA were read at this point. Standard calibration curves were generated by plotting C(t) values against log (arbitrary DNA concentration). This showed a linear correlation between C(t) and log(DNA concentration) with R^2 ^value >0.95. The arbitrary DNA concentration of each sample was calculated using equations generated from the calibration curves. The resulting DNA concentration of samples was normalized against input and enrichments for histone modifications are presented as percentage input (1/20). The details of the antibodies and primers used in the ChIP assays are shown in Additional file 2, Tables S1 and S2.

### Restriction enzyme digest/Metabisulfite sequencing

DNA was isolated from cells using a standard phenol-chloroform extraction and ethanol precipitation protocol. For methylation-sensitive restriction digest, DNA was digested with *Bgl*II and *Hha*I (New England Biolab). Completion of genomic DNA digestion was controlled with λ DNA (or a plasmid) digestion mixed with each reaction mix. Digested genomic DNA was analysed by Southern blotting using a [^32^P]dCTP-labelled hCD2 probe and analysed using a Phosphorimager (Amersham Pharmacia) and Image Quant software (Molecular Probes). For metabisulfite sequencing, DNA was digested with *Afl*II and/or *Xba*I. Digested DNA was denatured by heating and treatment with NaOH and then treated with sodium bisulfite (Sigma). Nested PCR was performed using an Expand High Fidelity PCR kit (Roche) to amplify fully converted DNA templates. PCR products were cloned into a TA-cloning vector (Invitrogen). Approximately 30 clones were sequenced to determine the methylation status for each sample. Only the clones that showed >97% C->T conversion at non-CpG sites were used for analysis.

### DNase I hypersensitivity assay

DNase I hypersensitivity assay was carried out as described previously [18,31,42]. The extent of DNase I digestions were determined by 0.7% agarose gel electrophoresis of digested DNA samples.

### 3D-FISH

For 3D FISH, 1-2 × 10^5 ^cells were stuck to poly-L-lysine-covered coverslips and fixed with 0.1% glutaraldehyde in fix/permeabilization buffer (20 mM KH_2_PO_4_, 130 mM NaCl, 20 mM KCl, 10 mM EGTA, 2 mM MgCl2, 0.1% Triton-X100) for 30 min at RT. Fixation was quenched by treatment with Na borohydride. Cells were re-fixed with a mixture of EGS and sulfo-EGS before *in situ *denaturation of genomic DNA. hCD2 probe and γ-satellite probe were labelled using digoxigenin (DIG)-Nick Translation Mix (Roche) or with fluorescein-dUTP using Nick Translation Mix (Roche), respectively. Labelled probes were mixed with mouse-Cot1 DNA and salmon sperm DNA in FISH hybridization mix (50%(v/v) formamide, 2 × SSC, 10% dextran sulfate, pH7.2). Genomic DNA was denatured *in situ *by treatment with CAPS:NaOH solution (pH12.85-13.10) for 2 min and hybridized with heat-denatured probes for overnight-48 h at 38°C in a humidified chamber. Cells were washed extensively with 1-2× SSC/0.05% Tween-20. Signal from DIG-labelled hCD2 probe was visualized by treatment with sheep anti-DIG antibody (GIBCO), followed by rhodamine-conjugated donkey anti-sheep IgG antibody (Jackson Laboratories). After washes with 4 × SSC/0.05% Tween-20, cells were stained with DAPI (100 ng/ml) and mounted in Vectashield. Images were acquired and analysed using a DeltaVision System and Soft RoRx software (Applied Precisions). The experiments were conducted in a blind manner.

### Cryo-FISH

Ultracryosectioning and FISH was performed essentially as described in [47]. Resting T cells from hCD2 1.3B lymph nodes were fixed in 4% and 8% paraformaldehyde in 250 mM HEPES (pH 7.6; 10 min and 2 h, respectively) [68]. Cell pellets were embedded in 2.1 M sucrose in PBS and frozen in liquid nitrogen. Cryosections were cut using an UltraCut UCT 52 ultracryomicrotome (Leica, Milton Keynes, UK) with 200 nm in thickness, captured in sucrose drops, and transferred to coverslips for cryo-FISH.

Probes used to label pericentromeric clusters (γ- satellite plasmid; from Dr N Dillon) and human CD2 (hCD2-cos1 construct; from Dr D Kioussis) were labelled with DIG and biotin by nick translation (Roche), respectively, and separated from unincorporated nucleotides using microBioSpin P-30 chromatography columns (BioRad, UK). Hybridization mixtures contained 50% deionized formamide, 2 × SSC, 10% dextran sulphate, 50 mM phosphate buffer (pH 7.0), 1 μg/μl human Cot1 DNA, 2 μg/μl salmon sperm DNA, 1 μl nick-translated γ- satellite and 4 μl hCD2-cos1 DNA (in 6 μl hybridization buffer). Probes were denatured at 70°C for 10 min, and reannealed at 37°C for 30 min before hybridization. Cryosections were rinsed (three times in PBS, incubated (30 min) in 20 mM glycine in PBS, rinsed (three times) in PBS, permeabilized (10 min) with 0.2% Triton X-100 +0.2% saponin in PBS, and then washed (three times) in PBS. After washing with PBS, cryosections were incubated (1 h, 37°C) with 250 μg/ml RNase A, treated (10 min) with 0.1 M HCl, dehydrated in ethanol (50% to 100% series, 3 min each), denatured (12 min, 80°C) in 70% deionized formamide, 2×SSC and dehydrated as above. Hybridization was carried out at 37°C in a moist chamber for >40 h. Posthybridization washes were as follows: 50% formamide in 2×SSC (42°C; three times over 25 min), 0.1× SSC (60°C, three times over 30 min) and 4×SSC with 0.1% Tween-20 (42°C, 10 min). Sections were incubated (30 min) with casein-blocking solution (Vector Laboratories) containing 2.6% NaCl, 0.5% BSA, 0.1% fish skin gelatin, pH 7.5-8.0). The γ-satellite signal was amplified (2 h) with sheep Fab fragments anti-DIG (Roche), washed (three times over 1 h), incubated (1 h) with Cy3-conjugated donkey antibodies anti-sheep IgG (Jackson Laboratories). The hCD2 signal was amplified (1 h) with AlexaFluor488 Neutravidin (Molecular Probes, Eugene, OR), washed (three times over 1 h), incubated (1 h) with biotinylated goat anti-avidin antibodies (Vector), washed (three times over 1 h), incubated (1 h) with AlexaFluor488 Neutravidin, all in casein-blocking solution. Nuclei were counterstained with 2 μM TOTO-3 (Molecular Probes) in PBS/0.05% Tween-20, washed (four times) in PBS and mounted in VectaShield.

Hybridization of non-transgenic cells with hCD2 probes showed no detectable signals.

Images (TIFF files) were acquired on a confocal laser scanning microscope (Leica TCS SP2; 100× objective, NA 1.4, Milton Keynes, UK), equipped with Argon (488), HeNe (543 nm) and HeNe (633 nm) lasers, and pinhole equivalent to 1 Airy disk. Images (TIFF files) from the different channels were collected sequentially to prevent bleedthrough.

### Statistical analysis

Students *t*-test (unpaired, two-tailed), Fisher's exact test and Chi-squared test were carried out using GraphPad Software http://www.graphpad.com and Mann-Whitney's U-test was performed using VassarStats http://faculty.vassar.edu/lowry/utest.html. Also, non-parametric Kolmogorov-Smirnov test was carried out using an online calculator http://www.physics.csbsju.edu/stats/KS-test.html. Statistical methods used in particular analyses are stated in the main text or figure legends.

## Abbreviations

Ac: acetylation; APC: allophycocyanin; CFSE: carboxyfluorescin diacetate succinimidyl ester; ChIP: chromatin immunoprecipitation; DAPI: 4',6-diamidino-2-phenylindole; DIG: digoxigenin; EDTA: ethylenediamine tetraacetic acid; EGTA: ethylene glycol tetraacetic acid; FACS: fluorescence-activated cell sorter; FISH: fluorescence in *in situ *hybridization; H3K9: histone H3 lysine 9; hCD2: human CD2; HEPES: 4-(2-hydroxyethyl)piperazine-1-ethanesulfonic acid; HMTase: histone methyltransferase; HP1: heterochromatin protein 1; HSS: hypersensitivity site; IAP: intracicternal A particle; IP: immunoprecipitation; LCR: locus control region; LTR: long terminal repeats; me3: trimethylation; PBS: phosphate buffered saline; PCR: polymerase chain reaction; PE: phycoerythrin; PEV: position effect variegation; TC: tri-colour; TCRβ: T cell receptor β; TPE: telomeric position effect.

## Competing interests

The authors declare that they have no competing interests.

## Authors' contributions

KH-H participated in the project planning, carried out all the experiments and analyses except the cryo-FISH and the study on CD2 1.3-CTG mice, and drafted the manuscript. AP and SX performed the cyro-FISH and analyzed the data. AS carried out the study on CD2 1.3-CTG mice and helped to set up 3D-FISH. SUL was involved in ChIP and bisulfite sequencing experiments. RF was responsible for the overall design, interpretation of the results, coordination of the study and helped to write the manuscript.

## Supplementary Material

Additional file 1 **Figure S3**. Comparison of the level and distribution of histone marks between CD2 1.3A14 and CD2 1.3B hCD2- T cells. Click here for file

Additional file 2 **Supplementary tables**. Table S1 - List of the primers used in this study. Table S2 - List of the antibodies used in ChIP. Table S3 - Summary of 3D FISH results. Click here for file

Additional file 3 **Figure S1**. Analysis of the extent of T cell activation and proliferation during TCRβ/CD28 cross-linking. Click here for file

Additional file 4 **Figure S2**. hCD2- T cells from CD2 1.3-CTG transgenic mice exhibit an 'open' enhancer chromatin structure and a marked hCD2 derepression upon T cell activation. Click here for file
