# Supplementary material for: The molecular basis for stability of heterochromatin-mediated silencing in mammals
Source: Epigenetics Chromatin. 2009 Nov 4;2:14. doi: 10.1186/1756-8935-2-14 (PMC2779788; doi:10.1186/1756-8935-2-14)
Supplement: Additional file 1 — Figure S3. Comparison of the level and distribution of histone marks between CD2 1.3A14 and CD2 1.3B hCD2- T cells. [file 1756-8935-2-14-S1.pdf]

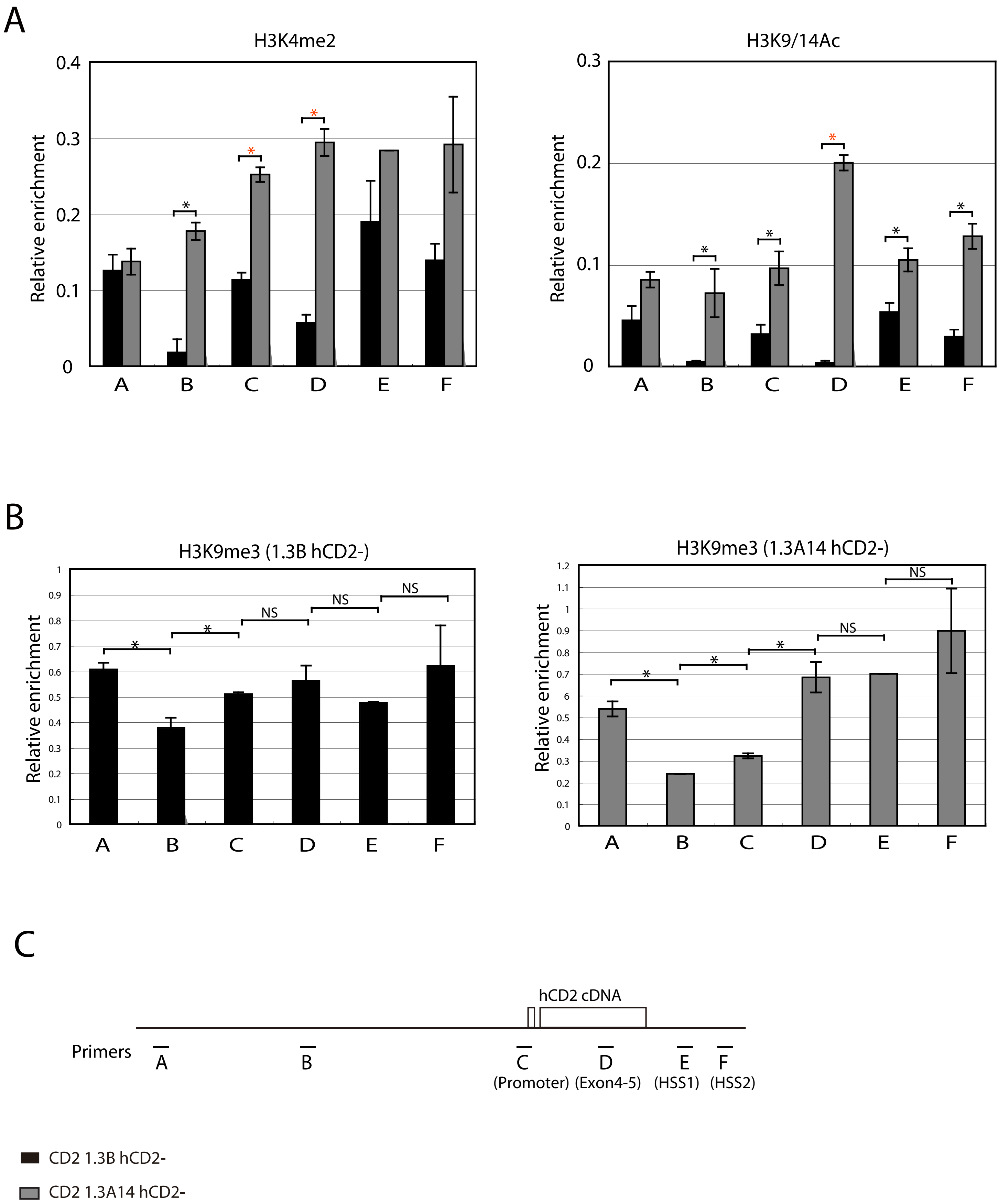
A
H3K4me2
Relative enrichment
0.4
0.3
0.2
0.1
0
A B C D E F
H3K9/14Ac
Relative enrichment
0.3
0.2
0.1
0
A B C D E F
B
H3K9me3 (1.3B hCD2-)
Relative enrichment
NS
A B C D E F
H3K9me3 (1.3A14 hCD2-)
Relative enrichment
NS
A B C D E F
C
hCD2 cDNA
Primers
A
B
C
(Promoter)
D
(Exon4-5)
E
(HSS1)
F
(HSS2)
CD2 1.3B hCD2-
CD2 1.3A14 hCD2-

Figure S3:

(A) Comparison of the H3K4me2 and H3Ac level along the hCD2 transgene locus between CD2 1.3B and CD2 1.3A14 hCD2- T cells. Black and grey bars represent enrichment of each histone modification in CD2 1.3B and CD2 1.3A14 hCD2- T cells respectively. Statistical significance of differences in the H3K4me2 and H3Ac level between the CD2 1.3B and CD2 1.3A14 transgenic mice is shown by asterisks (black asterisks = $p<0.05$, red asterisks = $p<0.005$ by Students t-test; error bars = S.D.). For the locations of region A-F, see (C) in this figure.

(B) Patterns of H3K9me3 differ between CD2 1.3B and CD2 1.3A14 hCD2- T cells. Black and grey bars represent enrichment of H3K9me3 in CD2 1.3B and CD2 1.3A14 hCD2- T cells respectively. The dip seen in K9me3 levels extends from A-C in the case of 1.3B CD2- cells whereas in CD2 1.3A14 – cells this dip extends further from A-D. Statistical significances of differences in the H3K9me3 level between ‘adjacent’ regions along the hCD2 transgene locus is shown by black asterisks ($p<0.05$; NS=not significant by t-test).

(C) Schematic diagram of hCD2 transgene locus. Locations of the primers used for ChIP assays are indicated with black bars and letters at the bottom.
