# Supplementary material for: The molecular basis for stability of heterochromatin-mediated silencing in mammals
Source: Epigenetics Chromatin. 2009 Nov 4;2:14. doi: 10.1186/1756-8935-2-14 (PMC2779788; doi:10.1186/1756-8935-2-14)
Supplement: Additional file 2 — Supplementary tables. Table S1 - List of the primers used in this study. Table S2 - List of the antibodies used in ChIP. Table S3 - Summary of 3D FISH results. [file 1756-8935-2-14-S2.doc]

**Table S1: List of the primers used in this study**

| Primer | Sequence | Tm(°C) |
| --- | --- | --- |
|  | ***Primers for ChIP*** |  |
| 337-586 (250/44) Fwd | GCTTTTACTGCCTCTGCTGGTTC | 63.1 |
| 337-586 (250/44) Rvs | AAGGTGGTAGAAGTTGGTTATTGCC | 65.8 |
| 2225-2538 (271/272) Fwd | TCAGCCACTCACAAACAGGAA | 65.8 |
| 2225-2538 (271/272) Rvs | AGAGTAGCTACCCGTGGAGACAG | 64.7 |
| Promoter Fwd | CACGTGGTTAAGCTCTCGGGG | 72.4 |
| Promoter Rvs | TCAGGGAAAGCTGGGTTGGGA | 71.9 |
| Exon 4-5 Fwd | GCCTCTTGATGGTCTTTGTG | 62.3 |
| Exon 4-5 Rvs | TCTGCTGGTGAACTTGTGTG | 62.7 |
| HSS1 Fwd | AAAGCTCCATGCACAAACGTT | 68.5 |
| HSS1 Rvs | TGCATGACTGCTAGCTGGGGAT | 70.0 |
| HSS2 Fwd | TCGGGAGGTTGAGGCAGAATA | 67.8 |
| HSS2 Rvs | AATGGCGTATGAGCTGTTCTGG | 67.1 |
| CD3ε promoter Fwd | CCTGTCTGCTTCTGGTGCCTTCTTC | 71.8 |
| CD3ε promoter Rvs | GAAAGTGTTCCACCGCATCCTCTCA | 72.9 |
| β-actin promoter Fwd | TCCGGGCCTCGATGCTGAC | 73.3 |
| β-actin promoter Rvs | TACCCCCTCTCCCCTCCTTTTGA | 71.2 |
| IAP (LTR) Fwd | tctgcgcatatgccgagggtgg | 76.7 |
| IAP (LTR) Rvs | ggatcttccttgcgccagtcccg | 76.9 |
|  | Primers for bisulfite sequencing |  |
| HSS Fwd | GTAGTTATGTAGTTGTATTTTGAGGGAAG | 61.1 |
| HSS Rvs | CTATTCTAATAAAATAACCAATAAAATCACTC | 59.8 |
| HSS Fwd (for nested PCR) | AGGGAAGTGGGGAGAAAAAAATA | 64.4 |
| HSS Rvs (for nested PCR) | TCTAATAAAATAACCAATAAAATCACTCATAAA | 61.8 |

| Antibody | Company | Catalogue No. | Lot No. | Reference |
| --- | --- | --- | --- | --- |
| H3K4me2 | Abcam | ab7766 | 134743 | Dover et al., 2002 Zhang et al., 2009 |
| H3K9/14Ac | Upstate | 06-599 | 23793 | Brumstein et al., 1996 |
| H3K9me1 | Dr. T. Jenuwein | N/A | 4548 | Peters et al. 2003 |
| H3K9me2 | Dr. T. Jenuwein | N/A | 4679 | Peters et al. 2003 |
| H3K9me3 | Dr. T. Jenuwein | N/A | 4861 | Peters et al. 2003 |
| IgG | Santa Cruz | sc-2027 |  |  |

**Table S2: List of the antibodies used in ChIP**

* We used several lots but we could obtain similar enrichments for the modifications.

Anti-H3K9me1/me2/me3 antibodies were kindly provided by Dr T. Jenuwein (IMP, Austria).

**Table S3: Summary of 3D FISH results**

|  | | **CD2 1.3A14 hCD2+** | | **CD2 1.3A14 hCD2-** | |
| --- | --- | --- | --- | --- | --- |
| **Not associated**  **with heterochromatin*^1, 2^** | | **74/89 (83.1%)** | | **39/154 (25.3%)** | |
| **Close proximity**  **to heterochromatin*^1^** | **Nuclear periphery*^2^** | **15/89**  **(16.9%)** | **3/89 (3.4%)** | **115/154**  **(74.7%)** | **68/154 (44.2%)** |
|  | **γ−satellite cluster*^2^** |  | **12/89 (13.5%)** |  | **47/154 (30.5%)** |

3D FISH was performed on sorted hCD2+ and hCD2- peripheral T cells from CD2 1.3A14 transgenic mice. The cells in which the transgene signal is located within less than 0.4μm from the nearest γ-satellite cluster/nuclear periphery are categorized as ‘close proximity to heterochromatin’. The rest was categorized as ‘not associated with heterochromatin’. The category ‘close proximity to heterochromatin’ could be further sub-divided into ‘(close proximity to) γ-satellite cluster’ or ‘(close proximity to) nuclear periphery’. The number (percentages) of the cells that fall into each category is shown in the table. The results of statistical tests are shown at the bottom of the table.

Between the CD2 1.3A14 hCD2+ and hCD2- T cells, differences in the proportions of the cell which fall into each of the above categories are statistically significant:

*^1^ p<0.0001 by Fisher’s exact test & by χ^2^ test

*^2^ p<0.0001 by χ^2^ test.

***References for supplemental tables***

Braunstein, M., Sobel, R. E., Allis, C. D., Turner, B. M., and Broach, J. R. 1996.

Efficient transcriptional silencing in Saccharomyces cerevisiae requires a heterochromatin histone acetylation pattern. *Mol Cell Biol* **16**: 4349-4356.

Dover, J., Schneider, J., Tawiah-Boateng, M. A., Wood, A., Dean, K., Johnston,

M., and Shilatifard, A. 2002. Methylation of histone H3 by COMPASS

requires ubiquitination of histone H2B by Rad6*. J Biol Chem* **277**: 28368-28371.

Zhang, X., Bernatavichute, Y. V., Cokus, S., Pellegrini, M., and Jacobsen, S. E. 2009.

Genome-wide analysis of mono-, di- and trimethylation of histone H3 lysine 4 in

Arabidopsis thaliana. *Genome Biol* **10**: R62.
