# Supplementary material for: The molecular basis for stability of heterochromatin-mediated silencing in mammals
Source: Epigenetics Chromatin. 2009 Nov 4;2:14. doi: 10.1186/1756-8935-2-14 (PMC2779788; doi:10.1186/1756-8935-2-14)
Supplement: Additional file 3 — Figure S1. Analysis of the extent of T cell activation and proliferation during TCRβ/CD28 cross-linking. [file 1756-8935-2-14-S3.pdf]

Hiragami_Hamda
FigS1

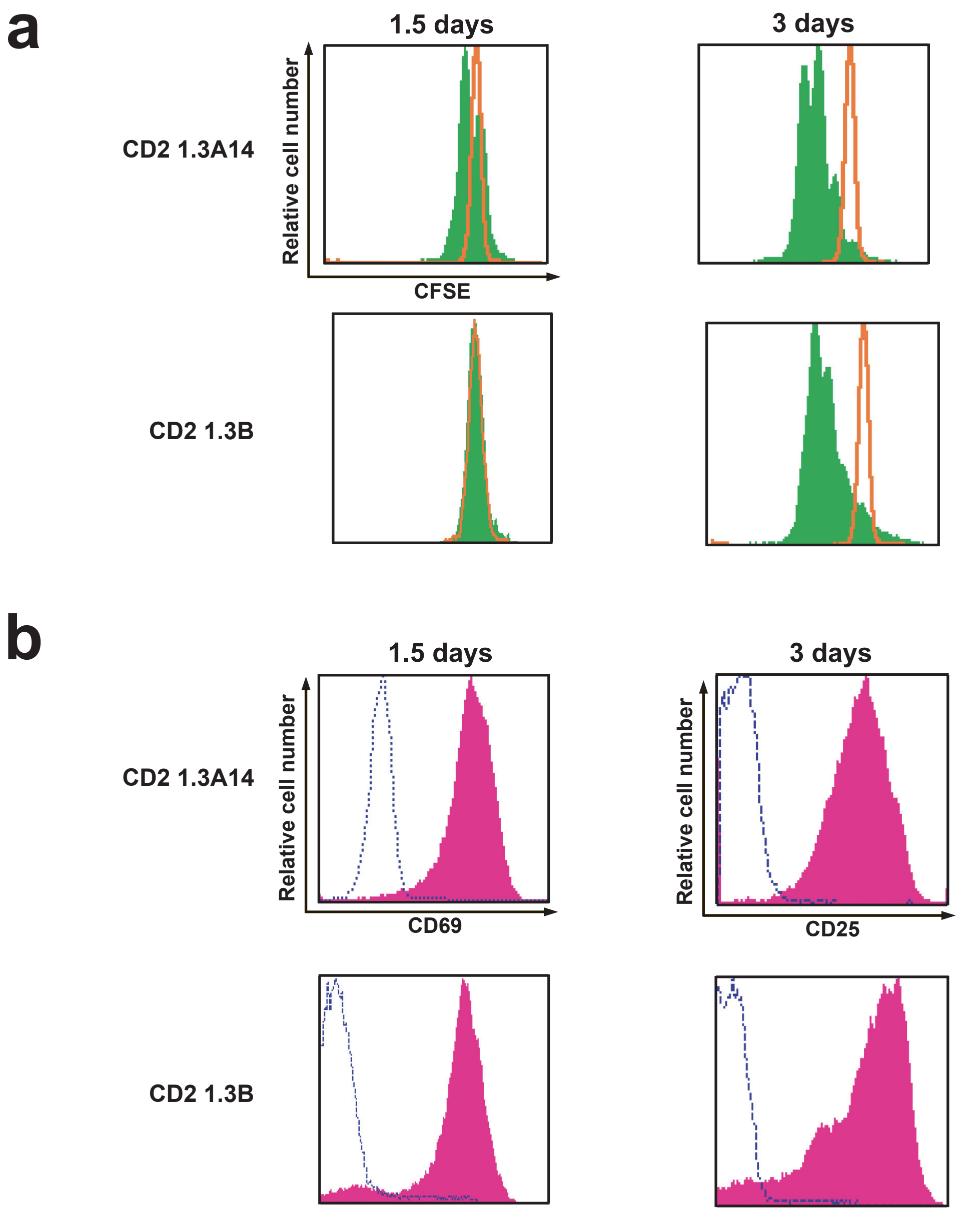

Supplementary figure 1

Analysis of the extent of T cell activation and proliferation during TCRβ/CD28 cross-linking.

(A) Cell proliferation profiles of hCD2- T cells from CD2 1.3B and CD2 1.3A14 transgenic mice. Sorted hCD2- T cells were loaded with CFSE prior to cell culture and incubated in the presence or absence of TCRβ/CD28 cross-linking for 1.5 or 3 days. The extent of cell proliferation was measured by mean of CFSE dilutions using FACS. In the histograms, the intensity of CFSE is plotted against cell numbers. The CFSE intensity of the activated T cells is plotted in solid green and that of the non-activated T cells are indicated with orange lines.

(B) Expression profiles of surface T cell activation markers on hCD2- T cells from CD2 1.3B and CD2 1.3A14 transgenics. Up-regulation of an immediate-early T cell activation marker, CD69 or another T cell activation marker, Interleukin-2 receptor α (CD25) on the surface of activated or non-activated T cells from CD2 1.3B and 1.3A14 transgenics were monitored at the indicated time-points by staining with fluorophore-conjugated antibodies against the activation makers and FACS. In the histograms, the intensity of CD69 (1.5 days) or CD25 (3 days) fluorescence is plotted against relative cell number. CD69/CD25 fluorescence of the activated T cells and that of the non-activated T cells are shown in solid pink or dotted blue lines respectively.
