# Supplementary material for: The molecular basis for stability of heterochromatin-mediated silencing in mammals
Source: Epigenetics Chromatin. 2009 Nov 4;2:14. doi: 10.1186/1756-8935-2-14 (PMC2779788; doi:10.1186/1756-8935-2-14)
Supplement: Additional file 4 — Figure S2. hCD2- T cells from CD2 1.3-CTG transgenic mice exhibit an 'open' enhancer chromatin structure and a marked hCD2 derepression upon T cell activation. [file 1756-8935-2-14-S4.pdf]

Hiragami-Hamada
FigS2

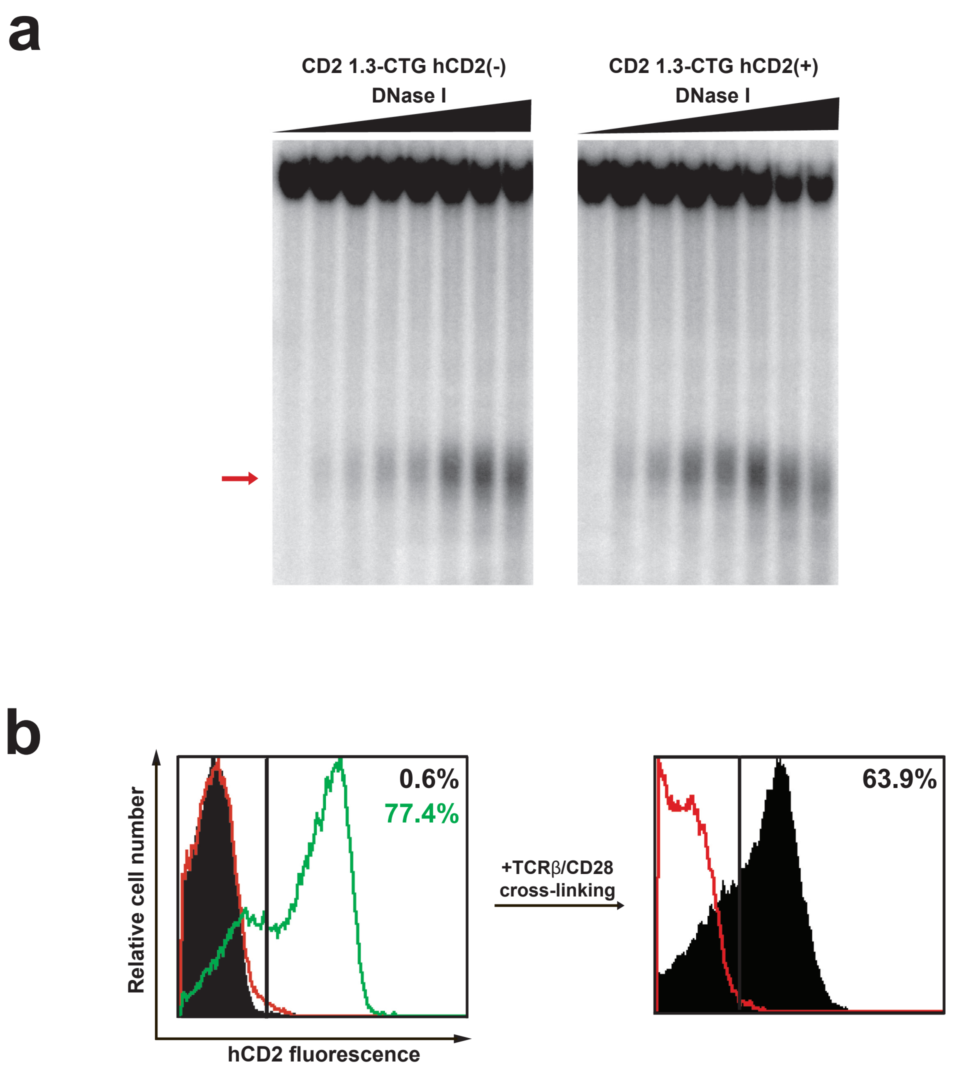

Supplementary figure 2

hCD2- T cells from CD2 1.3-CTG transgenic mice exhibit an ‘open’ enhancer chromatin structure and a marked hCD2 derepression upon T cell activation

(A) DNase I hypersensitivity assay on hCD2+ and hCD2- T cells from CD2 1.3-CTG transgenic mice. Nuclei isolated from hCD2+ or hCD2- T cells from the transgenic were treated with increasing concentrations of DNase I. DNA was isolated from the nuclei and accessibility of DNase I to the enhancer region of the hCD2 transgene was analysed by Southern blot. The transgene map and location of the probe are shown in Figure 4. The expected enhancer HSS is indicated with a red arrow.

(B) hCD2 derepression profile of hCD2- T cells from CD2 1.3-CTG transgenic mice during T cell activation. Sorted hCD2- T cells from the transgenic were activated by TCRβ/CD28 cross-linking for 3 days and changes in hCD2 expression were examined by FACS as described in Figure 5. In the histograms, hCD2 fluorescence is plotted against relative cell number. The left panel shows hCD2 expression profiles of unsorted (green line), sorted hCD2- (solid black) and non-transgenic (red line) T cells before T cell activation. The right panel shows hCD2 expression profile of ‘hCD2-‘(solid black) and non-transgenic (red line) T cells after 3 days of activation. The accompanying percentages indicate percentages of hCD2+ T cell population.
